# A text message intervention to support women’s physical and mental health after breast cancer treatments (EMPOWER-SMS): a randomised controlled trial protocol

**DOI:** 10.1186/s12885-019-5886-8

**Published:** 2019-07-04

**Authors:** A. Singleton, S. R. Partridge, R. Raeside, M. Regimbal, K. K. Hyun, C. K. Chow, K. Sherman, E. Elder, J. Redfern

**Affiliations:** 1 Faculty of Medicine and Health, Westmead Applied Research Centre (WARC), The University of Sydney, Westmead, NSW Australia; 2 Faculty of Medicine and Health, Sydney School Public Health, Prevention Research Collaboration, Charles Perkins Centre, The University of Sydney, Sydney, NSW Australia; 3 Centre for Emotional Health, Department of Psychology, Macquarie University, Sydney, NSW Australia; 4 Westmead Breast Cancer Institute, Westmead Hospital, Sydney, NSW Australia; 5 The George Institute for Global Health, Camperdown, NSW Australia; 6 Department of Cardiology, Westmead Hospital, Sydney, NSW Australia

**Keywords:** Breast cancer, Survivorship, Support, Text message, Self-efficacy, Randomised controlled trial, mHealth, Translational research

## Abstract

**Background:**

Breast cancer is the most common cancer diagnosed in women worldwide. In developed countries, 80–90% of women will survive five years after diagnosis but the transition from hospital-based care to health self-management and self-efficacy can be difficult. Text messaging programs offer a simple and proven way to provide support to people with chronic diseases. This study aims to test the effectiveness of a text message support program at improving women’s health self-efficacy, and physical and mental health outcomes after breast cancer treatments compared to usual care at 6-months and to understand the barriers and enablers to widespread implementation.

**Methods:**

Single-blind randomised control trial (RCT; *N* = 160) comparing a text message support intervention to usual care in women with breast cancer (recruited from a large tertiary referral hospital in Sydney, Australia). The intervention group will receive a six-month text message support program, which consists of semi-personalised, supportive, lifestyle-focused text messages (4 messages/week) in addition to usual care. The control group will receive usual care without the text message program. Outcomes will be assessed at 6-months. The primary outcome is change in self-efficacy for managing chronic disease. Secondary outcomes include change in clinical outcomes (body mass index), lifestyle outcomes (physical activity levels, dietary behaviours), mood (depression and anxiety scales), quality of life, satisfaction with, and usefulness of the intervention. Analyses will be performed on the principle of intention-to-treat to examine differences between intervention and control groups.

**Discussion:**

This study will test if a scalable and cost-effective text-messaging intervention is effective at improving women’s health self-efficacy, as well as physical and mental health outcomes. Moreover, this study will provide essential preliminary data to bolster a large multicentre RCT to helpsupport breast cancer survivors throughout recovery and beyond.

**Trial registration:**

Australia New Zealand Clinical Trials Registry (ANZCTR) number ACTRN12618002020268, 17 December 2018

## Background

Breast cancer is the most common cancer diagnosed in women worldwide [1]. Due to improvements in diagnostics and treatments in developed countries, 80–90% of women survive at least 5 years after their initial diagnosis [2]. During treatments, patients typically visit their doctors weekly and then gradually reduce to annual visits [3]. During this transition from hospital-based care to health self-management, breast cancer survivors are encouraged to exercise, maintain a healthy diet and weight [4], and, in the majority of cases, adhere to endocrine treatments [5] to reduce the risk of breast cancer recurrence. Many survivors find this transition physically and mentally difficult due to reduced interaction with, and psychological support from, medical professionals [6] and/or ongoing treatment side effects [5]. Therefore, simple and cost-effective strategies are needed to support breast cancer survivors during their recovery and beyond.

Self-efficacy is a person’s confidence with successfully completing a task. Self-efficacy can be improved through 1) learning information 2) motivating goal setting and self-evaluation and 3) achieving goals. Moreover, self-efficacy is domain specific, indicating that people can improve self-efficacy for one task, such as medications adherence, but it will not improve self-efficacy for another task, such as engaging in physical activity [7, 8]. Research shows that educating people about health self-management has been found to improve self-efficacy [7, 9–12], reduce inaccurate illness perceptions [9], drive behaviour change and improve health outcomes [7, 9–12]. However, education programs commonly involve in-person or phone-based counselling, which are labour and cost intensive [13, 14].

Mobile health (mHealth) is the use of mobile technologies, such as mobile phones and applications, to support healthcare delivery [15]. mHealth interventions are cost-saving compared to traditional education programs [16, 17] and align well with self-efficacy principles by providing encouragement to achieve health goals, improving health self-management skills through education and goal setting, and tracking progress and accomplishments [18, 19]. However, most studies target improvements in only one self-efficacy domain. For example, a pre-post study using an interactive weight-loss app found that improvements in eating self-efficacy was related to weight-loss in overweight/obese endometrial and breast cancer survivors [20]. Another study tested three apps, which used self-guided cognitive behavioural therapy to improve mental health, emotional self-awareness and coping self-efficacy compared to usual care and found that coping-self efficacy mediated improvements in depression scores for people who accessed a national mental health organisation website [21]. However, it is unclear if mHealth interventions that target multiple self-efficacy domains can promote improvements in various health outcomes simultaneously.

Text messages are currently the widest-reaching mHealth intervention, as they do not require the internet for delivery [22, 23]. Globally, there are more than 5 billion unique mobile phone users from urban and rural communities [23], and these users send more than 18 billion text messages per day [24]. Text-messaging has, therefore, been targeted as a simple tool for providing people with health information [22]. Recently, the Tobacco, Exercise and Diet Messages (TEXTME) randomised controlled trial (RCT) tested a lifestyle-focused text message intervention (4 messages per week for 6 months), which provided health advice, motivational reminders and support to change lifestyle behaviours compared to usual care for people with coronary heart disease who had recently been hospitalised. At six-months, results showed the TEXTME program effective for promoting healthy behaviours and improving clinical outcomes, such as low-density lipoprotein (LDL)-cholesterol, body mass index (BMI), rates of healthy eating and smoking cessation compared to usual care [25, 26]. Moreover, participants found the TEXTME program useful (91%) and motivating (77%) during their transition to health self-management [27]. For women with breast cancer, text message programs have been shown to help reduce weight, improve exercise frequency, adherence to endocrine therapies [28] and improve motivation to achieve health goals [29]. Although these studies used strategies that are known to improve self-efficacy, such as providing education and motivational information [7], they did not specifically investigate self-reported changes in self-efficacy.

The current study aims to improve self-efficacy in multiple domains related to chronic disease risk factors (physical activity, nutrition, social and emotional well-being, medication adherence, breast cancer knowledge) by providing educational, motivational and supportive information via text message during the difficult transition from hospital-based care. Therefore, this study aims to test the effectiveness of a six-month text message program [9] in addition to usual care and test its efficacy in improving women’s self-efficacy for managing chronic disease after breast cancer treatments compared to usual care alone. We also aim to determine if the text message program can improve anxiety, depression, BMI, healthy eating behaviours, physical activity and quality of life, and if self-efficacy mediates these improvements. Finally, this study aims to investigate the barriers and enablers to implementing such a program with women recovering from breast cancer treatments.

## Methods

### Study design

**Fig. 1:**
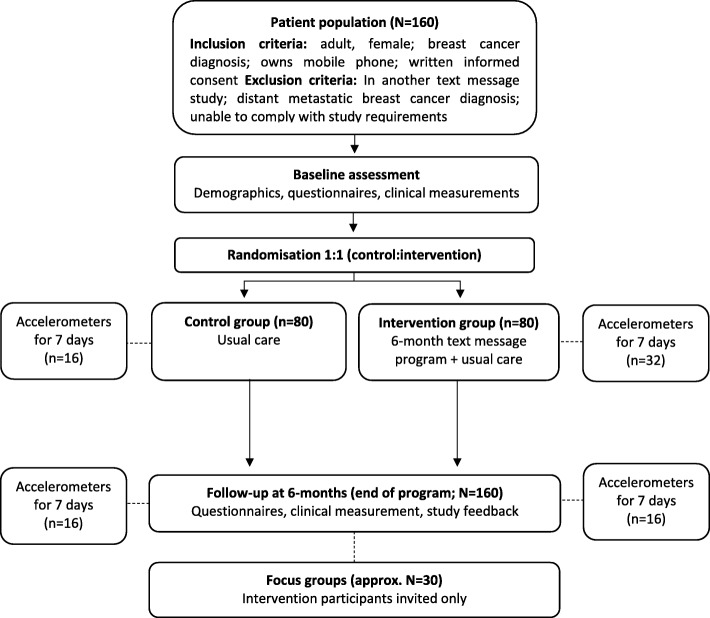
Study flow diagram. Eligible participants who agree to participate (i.e. sign consent form) are randomised to the intervention (text message program) or control (usual care) by a computerised programme. Baseline, follow-up and focus group assessments are performed

Single-blind RCT with a six-month follow-up comparing a text message support intervention to usual care in a population of women with breast cancer (Fig. 1). Written and informed consent will be obtained from all participants, and the study will follow the Consolidated Standards of Reporting Trials guidelines [30]. Clinical, behavioural and quality of life data will be collected at face-to-face assessments. To maintain blinding, randomisation will occur after the assessment via computer, which delivers an automated text message to participants regarding study groups but does not reveal allocation to the research assistant. Primary ethics approval has been received from the Western Sydney Local Health District Human Ethics Research Committee (AU RED HREC/18/WMEAD/281).

### Randomisation and blinding

After the baseline assessment, participants will be randomised to either usual care (control) or the text message intervention group in a uniform 1:1 (control:intervention) allocation ratio, using a secured central computer-based randomisation service. For each participant, the computer system automatically produces a study identification number, which will be used on all study documents. Within 7 days after the enrolment visit, the computer system automatically starts sending the text message program to the participant. Therefore, the researcher conducting baseline and six-month face-to-face assessments remains blinded to treatment allocation. As an additional precaution and for safety purposes, there will be an independent, unblinded researcher monitoring all incoming text messages.

To validate the self-reported physical activity questionnaire, a subset (32/160) of participants, will be randomly assigned to wear a small (matchbox sized) accelerometer for 7 days [31] at baseline and follow-up and return it to a member of the research team.

### Study population

Patients will be eligible to participate if they are female; > 18 years old; diagnosed with breast cancer; have completed breast cancer treatments (surgery, and/or chemotherapy and/or radiation therapy) within the past 18 months (although can still be on endocrine treatment); own an operational mobile phone capable of sending and receiving text messages; and, provide written informed consent. Patients will be excluded if they: are already participating in a text message-based study; have been diagnosed with distant metastatic breast cancer or cannot comply with study requirements (i.e., does not want to return for a follow-up visit, does not want to wear accelerometer). For those people who are ineligible or decline to participate, we will keep a ‘screening log’ of basic demographic information and reasons for non-participation.

### Study groups

*The usual care (control) group* will continue to receive usual care from their assigned health professionals, including support from breast care nurses and other allied health professionals. Participants in the usual care group will receive an initial text message welcoming them to the study, but they will not receive the text message support program. The control group will also receive a text message reminder approximately six-months after their enrolment, notifying them that they will be contacted to schedule their six-month follow-up visit. Participants in the control group will be offered the opportunity to receive the intervention at the end of the study if they wish.

*The intervention group (intervention)* will receive a text message support program in addition to usual care, where they will receive four messages per week at random times and days for 6 months. Participants will be informed that this is a one-way message program and not to reply. However, in case of replies we have a successful system (software program and a centrally located health counsellor with a clinical background) for monitoring all reply messages for safety. The messages will be semi-personalised, where some contain the participant’s preferred name and are tailored for individual circumstances and preferences (e.g., taking endocrine treatments or not). The messages will be sent from our team’s purpose-built and centralised web-based platform that provides simple registration and automated message delivery [32].

The message program content and structure was developed according to our previously published model [33], which involves a multiphase process of identifying breast cancer clinicians and survivors, hosting interactive workshops, and developing relevant and practical content, grounded in evidence-based guidelines and behaviour change theories and women’s lived experiences with breast cancer survivorship. The text message content (max. 100–250 characters) will provide support through education, practical health advice for specific and manageable behaviour change, motivational reminders for setting simple goals, and self-monitoring of goal achievement related to self-efficacy for managing chronic disease. Also, the messages strive to reinforce the initial advice and counselling that the patients received in the hospital. We used a patient-centred approach to develop messages relevant to women with breast cancer and common challenges of breast cancer survivorship [34]. The following message themes emerged: 1) general breast cancer information; 2) physical activity and nutrition; 3) medication adherence and symptom management; and, 4) emotional and social support and well-being.

Each week, messages will be selected from the message bank by the software system based on pre-specified algorithms that ensure that participants are receiving the correct messages (intervention vs control) and that the intervention group receives a variety across the four message themes. Both intervention and control groups will receive a welcome message at the beginning of the study and a concluding message at the end of the 6 months. All participants will receive training on how to read, reply, save and delete text messages. All participants will also be provided with the research personnel’s contact details and will be contacted at least once during the intervention period to facilitate the six-month follow-up interview. A researcher will manage a study mobile phone, and a record will be kept of any incoming messages from participants and all out-going replies from the study health counsellor throughout the study. Any analyses of these incoming text messages will be performed at the group level, except for reporting examples of individual quotes, which will be anonymised to protect the participant’s identity. Participants from either group can withdraw from the study at any time without giving a reason by replying ‘STOP’ to any of the messages or contacting a member of the research team, which will activate a process of review and withdrawal from the study. If a participant withdraws, no further information will be collected.

### Data collection

Patients will be recruited from the Westmead Breast Cancer Institute, a culturally and ethnically diverse breast cancer institute in Western Sydney, New South Wales, Australia. Potential participants will be identified through physician-referral, self-referral or researchers screening past and present patient appointment lists and invited to participate. If the patient has met all inclusion criteria and none of the exclusion criteria and she has provided written, informed consent to participate, she will be enrolled in the study by a researcher.

The researcher will collect baseline participant information during a face-to-face visit at the Breast Cancer Institute, such as demographics and physical measurements (eg. BMI, body fat percentage) and participants will complete several questionnaires to assess self-reported quality of life, depression, anxiety and physical activity (see Table 1). Participants will be asked information regarding their preferred method of receiving health information and if they currently use any technologies to help them with their health, including breast cancer-specific applications. Past medical history, a list of current prescription medications and chronic disease risk factors will be collected from the participant’s medical file.

**Table 1:** Study procedures and outcome measures collected at baseline and/or six-month follow-up assessments

	Baseline	Six-months (+/− 4 weeks)
Informed Consent	✓	
Inclusion/Exclusion Criteria	✓	
Demographics	✓	
Medical history (from hospital file)	✓	
Medication adherence (self-reported)	✓	✓
Accelerometer provided (*n* = 32)	✓	✓
Primary outcome		
Self-efficacy - Self-Efficacy to Manage Chronic Disease Scale (6-items) [35]	✓	✓
Secondary outcomes		
BMI measured by height in cm by stadiometer and weight in kilograms [36]	✓	✓
Total body fat percentage measured with Seca medical Body Composition Analyser (mBCA) 515 with Seca Analytics mBCA 115 software (Seca GmbH & Co. KG, Hamburg, Germany) [37]	✓	✓
Waist circumference in cm [36]	✓	✓
Physical activity level – GPAQ, a validated measure of mild, moderate and vigorous intense activity, as well as sedentary behaviours [38].	✓	✓
Nutrition - Questions adapted from WHO STEPS [39, 40]	✓	✓
Quality of life – measured by a core 30-item quality of life questionnaire, EORTC QLQ-30 AND	✓	✓
23-item breast cancer-specific sub-scale EORTC QLQ-BR23, which have been widely used and validated in breast cancer populations [41]	✓	✓
Illness perceptions – BIPQ, a validated 9-item measure used to rapidly assess patients’ perceptions of the cognitive and emotional impacts of their illness, including disease severity and causality [42].	✓	✓
Depression and Anxiety - short-form DASS, which has been validated and tested in numerous patient populations [43]. This scale will be collected from participants’ medical file, unless the scale was completed more than one month prior to the study visit, then participants will complete the scale.	✓	✓
Randomisation	✓	
Participant feedback survey		✓

*BMI* Body Mass Index, *WHO STEPS, World Health Organisation* STEPwise approach to chronic disease risk factor surveillance; *EORTC QLQ-30* European Organization for Research and Treatment of Cancer Quality of Life Questionnaire–Core; *EORTC QLQ-BR23* Breast Cancer subscale; *GPAQ* Global Physical Activity Questionnaire; *BIPQ* Brief Illness Perception Questionnaire; *DASS* Depression Anxiety Stress Scale

Six months after baseline, participants will be contacted to attend a face-to-face follow-up visit at the Breast Cancer Institute. Participants will be encouraged not to discuss whether they are receiving the text messages or not with the blinded researcher conducting the assessment. During this visit, researchers will repeat some of the same measurements as collected during the baseline visit, including the physical measurements and several questionnaires to assess self-reported quality of life, depression, anxiety and physical activity (see Table 1). Participants will also be asked about occurrences of any clinical events, healthcare visits and hospitalisations as well as their preferred method of receiving health information and if they currently use any technologies to help them with their health, including breast cancer-specific applications. A list of current prescription medications will be obtained from the participant’s medical file or in person, depending on availability. Finally, intervention participants will answer a survey regarding the acceptability, feasibility and utility of the text-messaging program. All data will be collected either on paper or electronic case report forms and will be monitored for quality assurance according to a pre-specified monitoring plan.

### Data confidentiality and storage

With the participants’ consent, the research staff will collect personal information about participants in-person and via participants’ medical files, which will only be used for the purpose of this research project and will only be disclosed with the participant’s permission, except as required by law. All paper data (excluding a confidential contact form – name, DOB, mobile number, email) will be quality checked against patient files then entered into a secure password-protected server-based database, which automatically checks data ranges. Information from the contact form will be entered into an enrolment log on a secured, password-protected hospital computer. Only study researchers will have access to study data and results that will be held securely in a locked filing cabinet or on a password-protected computer in Westmead Hospital. Paper and electronic data, including the consent forms and questionnaires, will be securely destroyed (paper documents shredded, electronic data and backups permanently erased) 5 years after study results are published.

### Outcomes

The primary outcome is self-efficacy for managing chronic disease (Table 1). Secondary outcomes (Table 1) include clinical measures (BMI, body fat percentage), lifestyle measures (physical activity, nutrition), quality of life, illness perceptions, depression and anxiety and medication adherence (self-report). We will also be collecting process measures to determine the barriers and enablers to providing a text message support program to breast cancer survivors. Participants will indicate their preferred method of receiving health information and rate the usefulness and appropriateness of the text message intervention. Any incoming text messages from participants throughout the intervention will be coded and analysed.

### Process evaluation

In addition to the stated outcomes, a log will be kept of the times the text messages were sent and what proportion were delivered successfully. Moreover, a log will be kept of the number of times the participants attempted to contact the research team and the method of contact (i.e. via phone, email, text message). At the end of the blinded follow-up assessment at six-months, intervention participants will complete a study questionnaire with Likert-scaled questions to examine the acceptability and utility of the intervention. This questionnaire will include items that address the acceptability of repeated text messages, identification of which messages the participants remembered, liked or disliked, what they did with the messages (i.e., saved, shared or deleted), the perceived utility of the text messages and opinions regarding the intrusiveness, timing and content suitability of the text messages.

A subset of intervention participants will also be invited in person or via telephone to participate in a focus group to provide a more in-depth understanding of the perceived barriers and facilitators to the intervention. These participants will be purposefully selected to diversify the opinions and views shared within the group by choosing participants with ethnically, culturally and socioeconomically diverse backgrounds. Each focus group will contain approximately 8–10 people and will last approximately 60 min. A minimum of two focus groups will be conducted and will be consecutive until no new themes or categories emerge (thematic saturation). It is anticipated that at least 16–20 participants will be invited to take part and will be reimbursed for their travel and parking expenses. The focus groups will be conducted by a trained interviewer, and a discussion guide will be used to facilitate and expand the discussion around key feedback themes such as utility and acceptability of the text messages and the perceived health impacts of the intervention. The focus groups will be digitally recorded and transcribed and coded into themes using NVivo version 11.0 (QSR International). The coded data will be interpreted, to isolate key themes, contrasts and hierarchies, and to develop coding matrices based on the Framework approach [44].

### Sample size and statistical analysis plan

A mean difference of 1 unit on the average score between patients in the intervention and control groups on the Self-Efficacy for Managing Chronic Disease Scale (6 items) would be a clinically meaningful improvement. A standard deviation of 2.05 for the Self-Efficacy for Managing Chronic Disease Scale (6 items) score (an average of the six items) was published in a similar cancer population [45]. With a 20% drop out rate, 80% power and type I error of 5%, a total of 160 patients will be needed, with 80 patients in each arm (intervention: control).

All analyses will be performed according to the intention-to-treat principle by a blinded statistician. Continuous variables will be summarised as means and corresponding 95% confidence intervals (CIs), or if the distribution is skewed, as medians and interquartile intervals. Categorical variables will be summarised as frequencies and percentages. Primary outcome will be compared between the intervention and the control groups at 6-months using analysis of covariance (ANCOVA), adjusting for the baseline measure of the primary outcome. Similarly, for each of the secondary outcomes, ANCOVA, adjusting for the baseline value of the outcome, will be used to compare between the groups at 6-months. We will also investigate if changes in secondary outcomes are mediated by self-efficacy scores. A significance level of 0.05 will be used. All analyses will be undertaken using SAS 9.4 for Windows.

## Discussion

This study aims to facilitate the transition from hospital-based care to health self-management for women with breast cancer by developing and testing a six-month lifestyle focused text message program in a RCT. This simple and cost-effective text message program uses positively-framed, informative and semi-personalised messages to motivate women to maintain healthy behaviours and remain connected with their oncology services. Expected results include improvements in primary and secondary outcomes. If successful, this study will inform a larger multi-centre RCT, with the goal of improving physical and mental health for breast cancer survivors during recovery and beyond. Results of the process measures will elucidate the barriers and enablers to widespread implementation of the text message program, with the ultimate goal of providing survivors with free, long-term out-of-hospital support after treatments.

## Conclusion

This study will test the implementation of a six-month text message program to support women’s physical and mental health after breast cancer treatments. If successful at improving health outcomes, this simple and cost-effective program can be easily scaled up to help breast cancer survivors nationally and internationally.

## Data Availability

Not applicable

## Abbreviations

BIPQ: Brief illness perception questionnaire
BMI: Body Mass Index
DASS: Depression Anxiety Stress Scale
EORTC QLQ-30: European Organization for Research and Treatment of Cancer Quality of Life Questionnaire–Core
EORTC QLQ-BR23: European Organization for Research and Treatment of Cancer Quality of Life Questionnaire - Breast Cancer subscale
GPAQ: Global Physical Activity Questionnaire
LDL: Low-density lipoprotein
RCT: Randomised controlled trial
TEXTME: Tobacco, exercise and diet messages study
WHO STEPS: World Health Organisation STEPwise approach to chronic disease risk factor surveillance

## References

[1] Jemal A, Bray F, Center MM, Ferlay J, Ward E, Forman D (2011). Global cancer statistics. CA Cancer J Clin.

[2] Australian Institute of Health and Welfare & Cancer Australia 2012. Breast cancer in Australia: an overview. Cancer series no. 71. Cat. no. CAN 67. Canberra: AIHW.

[3] Khatcheressian JL, Wolff AC, Smith TJ, Grunfeld E, Muss HB, Vogel VG (2006). American Society of Clinical Oncology 2006 update of the breast Cancer follow-up and management guidelines in the adjuvant setting. J Clin Oncol.

[4] Maura M, Boyle P, La Vecchia C, Decarli A, Talamini R, Franceschi S (1998). Population attributable risk for breast cancer: diet, nutrition, and physical exercise. J Natl Cancer Inst.

[5] Hershman DL, Shao T, Kushi LH, Buono D, Tsai WY, Fehrenbacher L (2011). Early discontinuation and non-adherence to adjuvant hormonal therapy are associated with increased mortality in women with breast cancer. Breast Cancer Res Treat.

[6] Lawler S, Spathonis K, Masters J, Adams J, Eakin E (2011). Follow-up care after breast cancer treatment: experiences and perceptions of service provision and provider interactions in rural Australian women. Support Care Cancer.

[7] Bandura A (1977). Self-efficacy: toward a unifying theory of behavioral change. Psychol Rev.

[8] Bandura A, Walters RH. Social learning theory. Englewood Cliffs: Prentice-hall; 1977.

[9] Petrie KJ, Cameron LD, Ellis CJ, Buick D, Weinman J (2002). Changing illness perceptions after myocardial infarction: an early intervention randomized controlled trial. Psychosom Med.

[10] Strecher VJ, McEvoy DeVellis B, Becker MH, Rosenstock IM (1986). The role of self-efficacy in achieving health behavior change. Health Educ Q.

[11] de Jongh T, Gurol-Urganci I, Vodopivec-Jamsek V, Car J, Atun R. Mobile phone messaging for facilitating self-management of long-term illnesses. Cochrane Database Syst Rev. 2012;(12):Cd007459. 10.1002/14651858.CD007459.pub2 PMC6486189 23235644

[12] Grembowski David, Patrick Donald, Diehr Paula, Durham Mary, Beresford Shirley, Kay Erica, Hecht Julia (1993). Self-Efficacy and Health Behavior Among Older Adults. Journal of Health and Social Behavior.

[13] Downe-Wamboldt BL, Butler LJ, Melanson PM, Coulter LA, Singleton JF, Keefe JM (2007). The effects and expense of augmenting usual cancer clinic care with telephone problem-solving counseling. Cancer Nurs.

[14] Bower PJ, Rowland N. Effectiveness and cost effectiveness of counselling in primary care. Cochrane Database Syst Rev. 2006;(3): CD001025. 10.1002/14651858.CD001025.pub2. 10.1002/14651858.CD001025.pub2 16855955

[15] Kay M, Santos J, Takane M (2011). mHealth: New horizons for health through mobile technologies. World Health Organ.

[16] Burn E, Nghiem S, Jan S, Redfern J, Rogers A, Thiagalingam A, et al. Cost-effectiveness of a text message programme for the prevention of recurrent cardiovascular events Heart. 2017;103:893-94. 10.1136/heartjnl-2016-310195 28235776

[17] Iribarren SJ, Cato K, Falzon L, Stone PW (2017). What is the economic evidence for mHealth? A systematic review of economic evaluations of mHealth solutions. PLoS One.

[18] Vollmer Dahlke D, Fair K, Hong YA, Beaudoin CE, Pulczinski J, Ory MG (2015). Apps seeking theories: results of a study on the use of health behavior change theories in cancer survivorship mobile apps. JMIR MHealth UHealth.

[19] Michie S, Abraham C, Whittington C, McAteer J, Gupta S (2009). Effective techniques in healthy eating and physical activity interventions: a meta-regression. Health Psychol.

[20] McCarroll ML, Armbruster S, Pohle-Krauza RJ, Lyzen AM, Min S, Nash DW (2015). Feasibility of a lifestyle intervention for overweight/obese endometrial and breast cancer survivors using an interactive mobile application. Gynecol Oncol.

[21] Bakker D, Kazantzis N, Rickwood D, Rickard N (2018). A randomized controlled trial of three smartphone apps for enhancing public mental health. Behav Res Ther.

[22] Cole-Lewis H, Kershaw T (2010). Text messaging as a tool for behavior change in disease prevention and management. Epidemiol Rev.

[23] GSMA Intelligence. The mobile economy; 2019. p. 1-56. Available from: https://www.gsmaintelligence.com/research/?file=b9a6e6202ee1d5f787cfebb95d3639c5&download.

[24] Burk K. How many texts do people send every day? 2016. Available at: http://www.trtest.xyz/blog/how-many-texts-people-send-per-day/.

[25] Chow CK, Redfern J, Hillis GS, Thakkar J, Santo K, Hackett ML (2015). Effect of lifestyle-focused text messaging on risk factor modification in patients with coronary heart disease: a randomized clinical trial. Jama..

[26] Thakkar J, Redfern J, Thiagalingam A, Chow CK (2016). Patterns, predictors and effects of texting intervention on physical activity in CHD–insights from the TEXT ME randomized clinical trial. Eur J Prev Cardiol.

[27] Redfern J, Santo K, Coorey G, Thakkar J, Hackett M, Thiagalingam A (2016). Factors influencing engagement, perceived usefulness and behavioral mechanisms associated with a text message support program. PLoS One.

[28] Mougalian SS, Epstein LN, Jhaveri AP, Han G, Abu-Khalaf M, Hofstatter EW, et al. Bidirectional text messaging to monitor endocrine therapy adherence and patient-reported outcomes in breast Cancer. JCO Clin Cancer Inf. 2017;(1):1–10. 10.1200/CCI.17.00015 30657377

[29] Job Jennifer R, Spark Lauren C, Fjeldsoe Brianna S, Eakin Elizabeth G, Reeves Marina M (2017). Women’s Perceptions of Participation in an Extended Contact Text Message–Based Weight Loss Intervention: An Explorative Study. JMIR mHealth and uHealth.

[30] Schulz KF, Altman DG, Moher D (2010). CONSORT 2010 statement: updated guidelines for reporting parallel group randomised trials. BMC Med.

[31] Neubeck L, Redfern J, Briffa T, Bauman A, Hare D, Freedman S (2008). The CHOICE (Choice of health options in prevention of cardiovascular events) replication trial: study protocol. BMC Cardiovasc Disord.

[32] Thakkar Jay, Barry Tony, Thiagalingam Aravinda, Redfern Julie, McEwan Alistair L, Rodgers Anthony, Chow Clara K (2016). Design Considerations in Development of a Mobile Health Intervention Program: The TEXT ME and TEXTMEDS Experience. JMIR mHealth and uHealth.

[33] Redfern J, Thiagalingam A, Jan S, Whittaker R, Hackett ML, Mooney J (2014). Development of a set of mobile phone text messages designed for prevention of recurrent cardiovascular events. Eur J Prev Cardiol.

[34] Cappiello M, Cunningham RS, Tish Knobf M, Erdos D (2007). Breast cancer survivors: information and support after treatment. Clin Nurs Res..

[35] Ritter PL, Lorig K (2014). The English and Spanish self-efficacy to manage chronic disease scale measures were validated using multiple studies. J Clin Epidemiol.

[36] Lohman TG, Roche AF, Martorell R. Anthropometric standardization reference manual. Champaign: Human kinetics books; 1988.

[37] Peine S, Knabe S, Carrero I, Brundert M, Wilhelm J, Ewert A (2013). Generation of normal ranges for measures of body composition in adults based on bioelectrical impedance analysis using the seca mBCA. Int J Body Comp Res.

[38] Bull FC, Maslin TS, Armstrong T (2009). Global physical activity questionnaire (GPAQ): nine country reliability and validity study. J Phys Act Health.

[39] Guthold R, Louazani SA, Riley LM, Cowan MJ, Bovet P, Damasceno A (2011). Physical activity in 22 African countries: results from the World Health Organization STEPwise approach to chronic disease risk factor surveillance. Am J Prev Med.

[40] v2.1 WSICaE (2008). The WHO STEPwise approach to chronic disease risk factor surveillance (STEPS).

[41] Sprangers M, Groenvold M, Arraras JI, Franklin J, te Velde A, Muller M (1996). The European Organization for Research and Treatment of Cancer breast cancer-specific quality-of-life questionnaire module: first results from a three-country field study. J Clin Oncol.

[42] Basu S, Poole J (2016). The brief illness perception questionnaire. Occup Med (Oxford, England).

[43] Siamak S, Bahram J, A study on the reliability and validity of the short form of the depression anxiety stress scale (DASS-21), Journal of social sciences and humanities of Shiraz University, 2007, Vol 26, Number 3 (52) (Special issue in education); Page(s) 65-77.

[44] Smith Joanna, Firth Jill (2011). Qualitative data analysis: the framework approach. Nurse Researcher.

[45] Foster C, Breckons M, Cotterell P, Barbosa D, Calman L, Corner J (2015). Cancer survivors’ self-efficacy to self-manage in the year following primary treatment. J Cancer Surviv.

